# Novel Treatments in Refractory Recurrent Pericarditis

**DOI:** 10.3390/ph17081069

**Published:** 2024-08-15

**Authors:** Emilia Lazarou, Christos Koutsianas, Panayotis K. Vlachakis, Panagiotis Theofilis, Dimitrios Vassilopoulos, Costas Tsioufis, George Lazaros, Dimitris Tousoulis

**Affiliations:** 1 First Cardiology Department, School of Medicine, Hippokration General Hospital, National and Kapodistrian University of Athens, Vas. Sofias 114, 11527 Athens, Greece; emilialaz@med.uoa.gr (E.L.); vlpanos@med.uoa.gr (P.K.V.); ptheofilis@med.uoa.gr (P.T.); ktsioufis@med.uoa.gr (C.T.); glaz35@hotmail.com (G.L.); 2 Clinical Immunology-Rheumatology Unit, 2nd Department of Medicine and Laboratory, Joint Academic Rheumatology Program, School of Medicine, National and Kapodistrian University of Athens, Hippokration General Hospital, 114 Vass. Sophias Ave, 11527 Athens, Greece; ckoutsianas@med.uoa.gr (C.K.); dvassilop@med.uoa.gr (D.V.)

**Keywords:** recurrent pericarditis, interleukin-1 blockers, pathophysiology, novel treatments, side effects

## Abstract

Refractory recurrent pericarditis is a troublesome condition that severely impairs the quality of life of affected patients and significantly increases healthcare spending. Until recently, therapeutic options included only a few medications and most of the patients resorted to chronic glucocorticoid treatment with steroid dependence. In the most recent decade, the introduction of interleukin-1 blockers in clinical practice has revolutionized the treatment of glucocorticoid-dependent and colchicine-resistant recurrent pericarditis due to their excellent efficacy and good safety profile. The rationale for the introduction of this class of medications in clinical practice is the autoinflammatory nature of recurrent pericarditis in a substantial rate of cases, with interleukin-1 being the main pro-inflammatory cytokine involved in this context. This review aims to discuss the contemporary available evidence from original research and real-world data on interleukin-1 blocker use in refractory recurrent pericarditis, in terms of indications, mechanism of action, efficacy, side effects, and recommended treatment protocols. Moreover, novel treatment proposals, such as hydroxychloroquine, *beta blockers*, and cannabidiol, which showed encouraging preliminary results, are addressed. Finally, gaps in knowledge, unmet needs, and future perspectives related to recurrent pericarditis are thoroughly discussed.

## 1. Introduction 

Recurrent pericarditis is a problematic pericardial syndrome in terms of patient management. Until approximately a decade ago, treatment options were very few and could be counted on the fingers of one hand. Indeed, treatment options included non-steroidal anti-inflammatory drugs (NSAIDs), colchicine, and glucocorticoids [1]. Additional treatments such as azathioprine, methotrexate, and intravenous human immunoglobulins were mostly used in autoimmune clinical phenotypes in the context of systemic diseases [1]. Data on their eventual benefits in idiopathic recurrent pericarditis are very scant and not robust [2,3]. 

In patients with long-lasting disease, which is not an uncommon possibility in several instances, most patients end up with a glucocorticoid-dependent status and are subjected to the detrimental adverse effects of chronic steroid use. The greater the dose of glucocorticoids required to maintain stable disease remission as well as the longer time to glucocorticoid exposure, the greater the impairment of the quality of life and the severity of treatment-related complications. 

Fortunately, the grey landscape of refractory recurrent pericarditis changed dramatically during the last decade and this is exclusively due to the introduction in clinical practice of a new class of medications called interleukin-1 (IL-1) blockers [4]. This new entry was based on the recent developments on recurrent pericarditis pathogenesis which, at least in a large proportion of cases, is ascribed to autoinflammation [5,6,7,8,9,10,11,12,13,14,15]. The latter includes a group of disorders attributed to a dysregulated innate immune system and the central cytokine responsible for the inflammatory reaction is IL-1 [9,15,16,17,18,19,20,21]. Notably, apart from IL-1 blockers, which at present are considered an established treatment in this setting, additional options have been proposed for the first time such as hydroxychloroquine and beta blockers, among others [22,23]. 

Although euphoria about IL-1 blocker use for the treatment of recurrent pericarditis is still prominent, several issues need further clarification. These include the identification of the ideal candidates for administration, treatment duration, need for dose tapering, and possible use as monotherapy or in conjunction with other medications [1,24].

In this review, we summarize the contemporary evidence on the use of novel treatments in troublesome patients with refractory recurrent pericarditis, namely glucocorticoid-dependent colchicine-resistant pericarditis with frequent (≥2) relapses at each attempt to reduce the dose of glucocorticoids below a certain threshold. In addition, gaps in knowledge and future perspectives are also discussed. 

## 2. Clinical Features 

Recurrent pericarditis is a troublesome and unpredictable disease. It complicates 15–30% of patients with a first episode of acute pericarditis [1]. Most importantly, patients who experience a first recurrence may enter into a vicious circle with several additional recurrences that complicate the course of the disease. Specifically, in the context of recurrent pericarditis, 5–10% of patients will develop the so-called colchicine-resistant glucocorticoid-dependent recurrent pericarditis, which is characterized by a high rate of relapses with a median disease duration that may be as long as approximately 5 years [25,26,27]. It is self-explanatory that refractory recurrent pericarditis has a detrimental impact on the quality of life of the affected patients who need repeated hospitalizations, emergency department visits, and diagnostic tests. However, the most frustrating aspect in similar cases is related to the most concerning side effects due to the chronic medical treatments and in particular to cortico-dependency. On the other hand, this series of events imposes a non-negligible cost for the health system, which cannot be ignored. 

Actually, for the diagnosis of recurrent pericarditis, we employ the same criteria required to establish the diagnosis of a first episode of pericarditis (see Table 1) [1,28,29,30,31,32]. At least two criteria should be present to establish the diagnosis [1]. Elevation of markers of inflammation and evidence of pericardial inflammation by an imaging technique constitute supportive findings [1]. It should be stressed that particular caution must be exercised when dealing with elderly subjects since the prevailing symptom in this specific population may be dyspnea instead of pleuritic-type chest pain [28]. Hospital admission is required in the presence of at least one of the high-risk features depicted in Table 1. The features in question have been associated with complicated pericarditis as well as with a higher rate of a specific etiology of acute pericarditis, which should be unveiled upon in-hospital work-up according to the clinical scenario [1]. Based on the presence of high-risk features, approximately 15% of patients diagnosed with pericarditis should be finally hospitalized [33].

In recent years, risk scores have been developed for the identification of patients prone to pericarditis recurrences or in general complicated pericarditis [34,35]. These scores have several characteristics in common such as the use of glucocorticoids, lack of colchicine administration, echocardiographic signs of constriction, and incomplete treatment of the target episode in terms of treatment duration, dose administered, and tapering plan. The introduction of these scores is an important step towards the improvement in the management of these unfortunate patients. Actually, patients classified as high risk for recurrences according to these classification systems should be treated based on optimal guideline-recommended treatment, such as administration of the maximal tolerated dose of NSAIDs, colchicine, implementation of a high threshold for glucocorticoids administration, and individualized tapering protocols based on serial C-reactive protein measurements to ensure that its plasma values are within normal limits before proceeding to dose tapering [1]. 

Another important issue that is worthy of discussion is that recurrent pericarditis is an umbrella term that includes a variety of clinical scenarios with different severity. For instance, some very problematic patients develop a recurrence during the tapering process in unacceptably high doses of glucocorticoids, e.g., at a dose equal to or higher than 25 mg of prednisone [36]. 

Another very concerning subgroup of patients is those with the incessant variant of recurrent pericarditis, namely those without a symptom-free interval of 4 to 6 weeks despite medical treatment. These patients depict a high rate of progression to pericardial constriction. This patient subgroup will probably benefit from third-level treatments with anakinra having emerged as the most valuable option in this setting [1,26]. 

On the other side of the clinical spectrum of recurrent pericarditis, there are patients who achieve stable disease remission with low and safe doses of glucocorticoids, which can be arbitrarily set below 5–7.5 mg of prednisone or an equivalent dose of an alternative glucocorticoid [37]. Indeed, below this threshold, glucocorticoids may be well tolerated so the cost/benefit ratio of IL-1 blockers may be questionable. In between the two edges of the spectrum, there are several scenarios and treatment decisions that should be individualized and extensively discussed with the patients. To summarize, from the general and barely informative term of recurrent pericarditis we should probably move to a slightly different terminology with the adoption of terms describing the specific scenario of the individual patient (high–moderate–low-severity recurrent pericarditis). Such an approach may have important implications in terms of treatment. 

Finally, in unfortunate cases with serious complications from pericarditis medical treatment, mainly from chronic steroid treatment, radical pericardiectomy constitutes the last resort. In centers with expertise in this type of intervention, pericardiectomy indeed is an overall safe procedure with good post-operative results [38]. 

## 3. Pathophysiology of Recurrent Pericarditis 

The pathophysiology of recurrent pericarditis remains controversial and a better understanding could significantly aid in disease management. Even though the majority of cases are characterized as “idiopathic”, there is increasing evidence that both autoimmune and autoinflammatory mechanisms play a crucial role. 

The inflammatory cascade frequently begins after an initial “hit” following a viral infection, but may also be related to bacteria, trauma, and irradiation, leading to initial damage to the pericardium. There are two main mechanisms of disease following this initial trigger, which may not always be easily distinguishable [39]. This trigger may cause a breach in adaptive immune tolerance providing autoantigens that activate B and T lymphocytes, stimulating the production of autoantibodies. The latter may be organ (anti-heart and anti-intercalated disk antibodies) or non-organ specific (anti-nuclear antibodies) and their presence has been well described in recurrent pericarditis [40], although their direct pathogenicity has not been proven and may be epiphenomena of the inflammatory process [41,42]. 

On the other hand, the initial tissue injury can also produce molecular patterns, either pathogen-associated molecular patterns (PAMPs) or damage-associated molecular patterns (DAMPs), which, in turn, activate the inflammasome via toll-like receptors (TLRs) and other cytokines, such as nuclear factor (NF)-κB and interferon-regulatory-factor-1 (IRF1) [43]. The formation and activation of the NLRP3 (NOD-, LRR-, and pyrin domain-containing protein 3) inflammasome mediates caspase-1 activation and subsequent release of pro-inflammatory cytokines, such as IL-1β and IL-18, as well as pyroptosis, which relates to rapid cell lysis and death [43]. This process is characteristic of a dysregulated innate immunity response with exaggerated IL-1 production, frequently characterizing autoinflammatory disorders, such as gout and periodic fever syndromes. Indeed, several recurrent pericarditis disease characteristics are reminiscent of these diseases, such as its relapsing nature, the highly elevated inflammatory reactants, the presence of fever, and serosal involvement. 

Based on all of the above, a distinction between different recurrent pericarditis clinical phenotypes has been proposed, a notion that may impact the choice of pharmacological treatment and general management [5,9]. The “autoinflammatory” phenotype is characterized by fever, serositis, and strikingly increased inflammatory markers and shows an outstanding response to IL-1 inhibition. The “autoimmune” phenotype shares many features with connective tissue diseases, such as Raynaud’s phenomenon, sicca syndrome, arthritis, photosensitivity, and autoantibody positivity, and generally has modest increases in erythrocyte sedimentation rate and C-reactive protein. Glucocorticoids, azathioprine, and hydroxychloroquine (HCQ), as well as colchicine, have shown satisfactory therapeutic results for this subgroup. Lastly, the third phenotype refers to patients with mildly symptomatic relapses, modest increases in acute phase reactants, and no connective tissue disease manifestations. For these patients, NSAIDs and colchicine may suffice to control the disease [1,22]. 

These insights in pathophysiology may guide the use of therapeutic agents in recurrent pericarditis. The majority of the therapeutic actions of glucocorticoids occur through their interaction with the glucocorticoid receptor. Their anti-inflammatory effects are mediated via changes in cellular proliferation, survival or differentiation, reduced expression of inflammatory mediators (mostly NF-κΒ and activator protein (AP) 1), or increased expression of anti-inflammatory factors [44]. Colchicine is effective in suppressing inflammation and preventing relapses of recurrent pericarditis [45]. It mainly acts by its ability to bind to tubulins and thus blocks the assembly and polymerization of microtubules, which are involved in various cellular processes. It therefore exerts its anti-inflammatory effect by disrupting neutrophil migration and downstream cellular functions of leucocytes while it also serves as a non-specific inhibitor of the inflammasome [46]. 

As suggested above, IL-1 is a key cytokine in the pathophysiology of recurrent pericarditis. Its two most important IL-1 family members, IL-1α and IL-1β, possess pro-inflammatory and regulatory properties and they bind to the same IL-1 receptor (IL-1R). IL-1α is released by the pericardial cells as an initial response to the triggering factor. IL-1α directly activates and recruits inflammatory cells and its effect is rather localized in the pericardium, where, besides inflammation, it may occasionally result in fibrosis and rarely constrictive pericarditis. IL-1α also acts as a stimulant for the production of IL-1β from monocytes. The latter is the main circulating form of IL-1 and serves as an amplifier of the inflammatory response, being responsible for the systemic effects of fever, serositis, and acute phase reactant elevation [15]. It thus appears to be important to block both IL-1α and IL-1β in order to achieve improved suppression of the inflammatory process and reduce relapses. 

At present, two IL-1 inhibitors able to neutralize both IL-1α and IL-1β are available for use in everyday clinical practice, namely anakinra and rilonacept. Anakinra is a recombinant human IL-1R antagonist, which by blocking the IL-1 receptor interferes with both IL-1α and IL-1β. Rilonacept is a dimeric fusion protein and it consists of the ligand-binding domains of the human IL-1R component (IL-1RI) and IL-1R accessory protein (IL-1RAcP) linked to the Fc portion of human immunoglobulin G1. As rilonacept enters circulation it acts as a soluble decoy receptor and “traps” IL-1α and IL-1β by binding to both and preventing their interaction with IL-1R on the cell surface. The pharmacokinetic profile of rilonacept has the advantages of slow subcutaneous absorption and a long elimination half-life and enables convenient weekly dosing, in contrast with the daily injectable anakinra use. IL-1 blockade has had proven efficacy in achieving sustainable remission in recurrent pericarditis while allowing rapid discontinuation of glucocorticoids and improving patient-reported quality of life [24]. 

**Figure 1:**
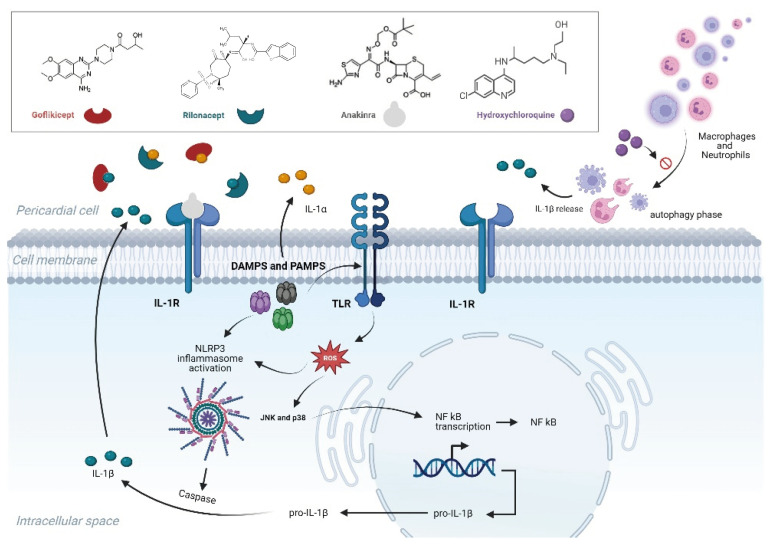
Mechanism of action of the novel drugs administered in glucocorticoid-dependent colchicine-resistant recurrent pericarditis (see text for details). IL-1 = interleukin-1, IL-1R = interleukin-1 receptor, DAMPs = damage-associated molecular patterns, PAMPs = pathogen-associated molecular patterns, TLRs = toll-like receptors, NLRP3 = NOD-like receptor protein 3 inflammasome, NF-κB = nuclear factor kappa-light-chain-enhancer of activated B cells, JNK = c-Jun N-terminal kinase, and ROS = reactive oxygen species. Created with BioRender.com.

Finally, another third-line drug recently employed as a steroid-sparing agent in refractory recurrent pericarditis is HCQ [22]. The latter medication is an antimalarial agent with immunomodulatory properties that have been established as treatment in several rheumatic diseases, such as systemic lupus erythematosus and rheumatoid arthritis. Despite it being in use for several decades, its mechanism of action remains not fully elucidated. HCQ accumulates within the lysosomes and impairs or inhibits lysosomal and autophagosome functions and subsequently immune activation. The process of autophagy blocked by HCQ is also involved in antigen presentation and immune activation, while its induction in neutrophils has recently been associated with pathogenic neutrophil extracellular traps (NETs) expressing IL-1β. Furthermore, hydroxychloroquine may interfere with TLR signaling and prevent TLR activation by changing endosomal pH. It has been shown that HCQ inhibits the production of several pro-inflammatory cytokines (such as IL-1, TNF, IFNγ, and IL-6) in monocytes as well as gene expression [47]. Based on the above observations, HCQ is a drug that may be efficacious in both the autoinflammatory and autoimmune clinical phenotypes of recurrent pericarditis. Figure 1 summarizes the pathophysiology of recurrent refractory pericarditis as well as the mechanism of action of the novel drugs used for its treatment.

## 4. Treatment 

### 4.1. Guideline-Recommended Treatment Algorithm 

**Figure 2:**
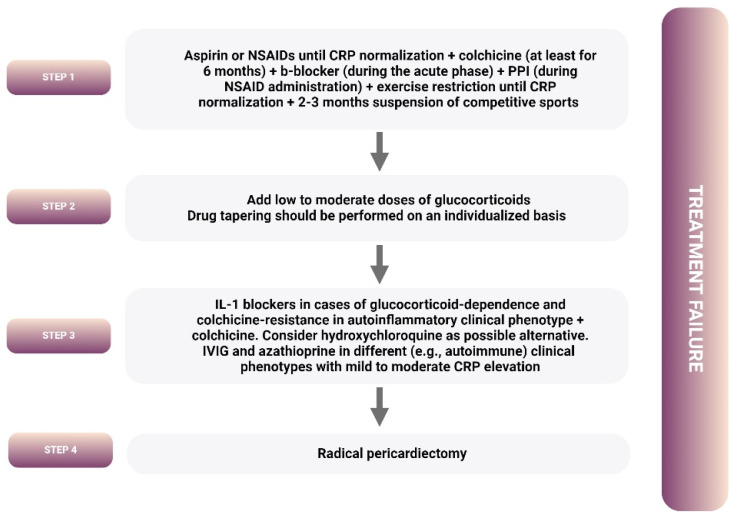
Recurrent pericarditis treatment algorithm according to the recommendations of the most recent 2015 European Society of Cardiology Guidelines for the diagnosis and management of pericardial diseases [1].

According to the most recent 2015 ESC Guidelines on pericardial diseases, the treatment approach of recurrent pericarditis includes NSAIDs plus colchicine as the first approach with the addition of low to moderate doses of glucocorticoids (triple therapy) as the second step in frequent and severe recurrences, or in the case of intolerance or contraindications to NSAIDs, in an effort to achieve disease control without remarkable side effects related to treatment (Figure 2) [1,48].

Colchicine was first introduced in the treatment of recurrent pericarditis in 1987 and constitutes an established option in the treatment of either the first or all subsequent episodes of pericarditis [49,50,51,52,53]. Specifically, colchicine has been shown to halve the rate of pericarditis relapses in patients with a first episode of recurrent pericarditis, in those with a first recurrence, and in those with multiple recurrences as well. 

Subsequently, the third-step approach in recurrent pericarditis is reserved for patients with at least two pericarditis relapses depicting colchicine resistance and steroid dependence, who as a rule suffer from devastating side effects from chronic glucocorticoid administration. The medications employed in this unfortunate patient subgroup include intravenous immunoglobulins, azathioprine, and IL-1 blockers with anakinra being the unique agent tested in clinical practice at the time of the 2015 Guidelines publication [1]. Apart from anakinra, however, data for the remaining agents are low quality and scant [2,3]. This review addresses novel treatments in acute pericarditis, with particular focus on IL-1 blockers.

### 4.2. IL-1 Blockers 

#### 4.2.1. Anakinra 

IL-1 blockers constitute the most important new entry that in recent years has revolutionized the management of refractory recurrent pericarditis. The first agent of this class that has been introduced in clinical practice is anakinra [54]. It was approved initially for the treatment of moderate to severe rheumatoid arthritis refractory to established treatments approximately 23 years earlier (in 2001) [55]. The first experience of anakinra in cases of recurrent pericarditis consisted of administration of the drug in three children with encouraging results in terms of efficacy and safety [4]. The above-mentioned results were confirmed 3 years afterward in three adults with refractory recurrent pericarditis [8]. The positive results observed in the case series prompted the design and conduction of the first randomized controlled trial of IL-1 blockade administration in idiopathic recurrent pericarditis, namely the AIRTRIP clinical trial [55]. This was a double-blind, placebo-controlled, randomized withdrawal trial with open-label administration of anakinra for 2 months. In patients with pericarditis resolution during the open-label period, a double-blind withdrawal step with placebo (10 patients) or anakinra (11 patients) for 6 months or earlier, until pericarditis recurrence, was applied. The presence of at least three pericarditis recurrences, C-reactive protein elevation at baseline, colchicine failure, and glucocorticoid dependence were all prerequisites for enrollment. Anakinra was administered subcutaneously at a dose of 2 mg/kg per day, up to 100 mg. The mean age of the study population was ~45 years and 4 out of 21 were female. All patients were followed up for 12 months while the median follow-up was 14 months. Pericarditis recurrence was observed in 90% of cases in the placebo group and in ~18% in the active medication group. Notably, the time to flare was 72 days in the placebo group whereas it was not possible to calculate this time in the anakinra group due to the low number of events. The most common side effect consisted of skin reactions and no permanent discontinuation of the active drug was recorded. 

AIRTRIP was the first study that, albeit small in size, showed for the first time an indisputable benefit of IL-1 blockade with a good safety profile in patients with recurrent pericarditis and an autoinflammatory clinical phenotype. The positive results presented in the latter trial were subsequently confirmed in the real-world IRAP registry [56]. This registry included 224 patients with a mean age of 46 years, consisting of 63% women. The most common pericarditis etiology (in 75%) was idiopathic, with an autoinflammatory clinical phenotype in the majority of cases, whereas the mean duration of the disease was 17 months. An important contribution of this registry was the delineation of the most efficacious treatment protocol with anakinra in terms of treatment duration and dose tapering protocol. Specifically, a full dose treatment period of at least 3 months followed by a tapering time interval of at least 3 months emerged as the therapeutic regimen associated with the lowest rate of recurrences. Regarding the endpoints after a median treatment of 6 months, pericarditis recurrences were reduced 6-fold, hospitalizations 7-fold, and emergency department visits 11-fold, while glucocorticoid use decreased from 80% to 27%. No serious adverse events were observed overall. Temporary skin reaction was the most commonly observed side effect (38%), with permanent discontinuation required in only 3% of cases. 

#### 4.2.2. Rilonacept 

A few years later, rilonacept, a new IL-1 blocker, was tested for the management of refractory recurrent pericarditis [57]. Rilonacept has the advantage of weekly subcutaneous administration instead of daily, which is important in terms of adherence to treatment and quality of life. The drug was tested in the RHAPSODY trial, which was a phase III multicenter, double-blind, event-driven, randomized withdrawal trial that was published 5 years after AIRTRIP [58]. The mean age of the study population was ~45 years, 57% were women, and the main etiology of pericarditis was idiopathic in 85% of cases, with the remainder consisting of post-cardiac injury syndrome. Notably, all patients had at least two recurrences and depicted autoinflammatory phenotype, with acute symptoms and C-reactive protein elevation. Similarly to AIRTRIP, the primary endpoint of this trial was the time to the first pericarditis recurrence, whereas safety issues were addressed as well. Rilonacept was given subcutaneously at a loading dose of 320 mg followed by 160 mg weekly. 

After a 12-week run-in period during which all pericarditis drugs were discontinued, 61 patients who showed a good clinical response according to prespecified criteria were randomized either to rilonacept given subcutaneously once a week or placebo. According to the results of this study, 74% in the placebo group depicted pericarditis recurrence, with the median time to first recurrence being 8.6 weeks. In the rilonacept group, pericarditis recurred in 7% of cases. The median time to the first recurrence could not be calculated in the rilonacept group due to the low number of events. With regard to safety, the most common adverse events consisted of injection site reactions and respiratory tract infections. To summarize, rilonacept in the RHAPSODY trial showed remarkable efficacy in terms of acute pericarditis resolution and prevention of relapses along with a good safety profile. After the publication of the above-mentioned results, the drug received FDA approval in March 2021 for the treatment of recurrent pericarditis and reduction in the pericarditis recurrence risk in adults and children 12 years and older. 

Notably, additional interesting RHAPSODY secondary analyses addressing different aspects of recurrent pericarditis were published as well. In particular, in a relevant analysis, the quality of life with the adoption of pertinent questionnaires during rilonacept treatment was assessed. This secondary analysis revealed an improved emotional and physical health of recurrent pericarditis patients while on medication. The observed improvements relate to patient-reported health-related quality of life, sleep quality, pain score, and overall symptom severity during rilonacept administration [59]. 

In an additional analysis of the long-term extension results of the RHAPSODY trial, it was shown that suspension of rilonacept administration after 18 months of treatment resulted in pericarditis recurrence in the great majority of cases, namely in six out of eight patients (75%) [60]. This finding confirms what has previously been observed in the real world with anakinra, namely that IL-1 blockade is extremely effective while on treatment, with pericarditis relapses appearing during discontinuation or dose tapering. Thus, although in recent years effective treatments have revolutionized the management of recurrent pericarditis, sadly medication that is able to cure the disease is not available so far. Unfortunately, according to preliminary data, it seems that not even a cardiac magnetic resonance-guided treatment strategy (based on the presence/absence of pericardial edema and pericardial late gadolinium enhancement) is able to identify patients that can discontinue rilonacept without risk of relapse [60]. 

The efficacy and safety of the above-mentioned IL-1 blockers (i.e., anakinra and rilonacept) were assessed in a recent metanalysis of seven studies including 397 patients (median age 42 years, 60% women) with recurrent pericarditis having an idiopathic etiology in 87% of cases [61]. In a median follow-up of 14 months, a significant reduction in pericarditis recurrences was observed compared to placebo (incidence rate ratio 0.06, 95% CI 0.03 to 0.14). Adverse events were more common with IL-1 blockers (risk ratio 5.38, 95% CI 2.08 to 13.92) and the risk ratio for infections was 3.65, 95% CI 1.23 to 10.85. 

#### 4.2.3. Goflikicept 

Interestingly a third IL-1 blocker has recently been administered for the treatment of refractory recurrent pericarditis in a small-sized investigation. The new IL-1 blocker employed in the latter phase II and III trials was goflikicept [62]. This drug is a heterodimeric fusion protein having a high affinity for IL-1α and IL-1β, which affects their signaling pathways. This study had an open-label, randomized, placebo-controlled design. Twenty patients (out of 22 initially enrolled during the run-in period) with idiopathic recurrent pericarditis were randomized [62]. The main advantage of this drug is the long period between (subcutaneous) administrations, which is 2 weeks. Goflikicept was administered at a dose of 160 mg at week 0, followed by 80 mg at weeks 1 and 2, and afterward 80 mg every 2 weeks. Pericarditis recurrence was observed in 9 out of 10 patients receiving placebo, while no recurrences were recorded in the active drug group within 24 weeks after randomization. Notably, no safety signals were identified. 

#### 4.2.4. Canakinumab 

Finally, another IL-1 blocker that has been administered in cases of refractory recurrent pericarditis is canakinumab [24]. The latter drug, canakinumab, binds to human IL-1β and neutralizes its inflammatory activity by blocking the interaction with IL-1 receptors [63]. In contrast, canakinumab does not have any effect on IL-1α or IL-1 receptor antagonists [24]. Data on canakinumab are very scant and include case reports and small case series [24,64,65,66]. The most important available data on its efficacy are controversial [24]. Therefore, as solid data are not currently available, canakinumab will not be further discussed in this review. 

#### 4.2.5. Adverse Reactions of IL1 Blockers 

Taking into consideration the aforementioned data, IL-1 blockers beyond any doubt are a valuable addition in the management of difficult-to-treat patients with refractory recurrent pericarditis. Regarding safety, both clinical studies and real-world evidence converge towards a definitely favorable risk/benefit ratio. Indeed, apart from hypersensitivity to the drug, which is the only absolute contraindication of IL-1 blocker administration, the remainder of adverse effects are in general transitory (e.g., skin reactions) and/or reversible (such as leucopenia, transaminasemia, and infections, among others), with either dose reduction or transient/permanent drug discontinuation [24,55,56,57,58,62]. The most common side effects along with their relevant frequencies of the currently in use IL-1 blockers are depicted in Table 2. Notably, treatment withdrawal due to drug-related serious side effects was deemed necessary in up to 3% of patients treated with anakinra or rilonacept [24].

In brief, in the context of IL-1 blockers used in recurrent pericarditis, most solid data are available for anakinra, which has been in use for >2 decades even if not exclusively for recurrent pericarditis [54]. Moderate experience has been gained in disease treatment with rilonacept, with relevant data stemming from its use on recurrent pericarditis after 2021 and from its previous use for alternative rare conditions such as Cryopyrin-Associated Periodic Syndromes and Muckle–Wells Syndrome [57,58]. Data on goflikicept are very poor at present and further evidence is required to delineate its safety [62]. 

Notably, most of the adverse reactions of IL-1 blockers are attributed to a class effect even though the rate of the individual complications may vary to a certain degree between the individual compounds. Injection site reactions are the most common side-effect of all IL-1 blockers [34]. They consist of erythema, occasionally painful, that may be attenuated or eventually prevented by warming the injection before use and application of ice packs locally for a few minutes after the injections. This side effect appears ~1–2 weeks after drug commencement and patients must be reassured about the transient nature of this adverse reaction since it disappears usually within 1 month. In more severe cases, local or systemic antihistamines or steroids may be given until resolution [24]. Neutropenia, transaminasemia, infections (most often of the respiratory tract), blood lipid elevation, arthralgias, and myalgias are additional side effects related to IL-1 blockade [24,56,57,58] (Table 2). Although the literature data regarding IL-1 blockers’ safety data are reassuring, their safety needs to be also confirmed in long-term studies in the specific setting of recurrent pericarditis. 

#### 4.2.6. IL1 Blockers in Specific Clinical Scenarios 

The use of IL-1 blockers has been a matter of concern in several clinical scenarios and subpopulations, for example, the administration of IL-1 inhibitors during conception and pregnancy. Although no safety signals have been detected in pregnant women, relevant data are insufficient, and informed decision-making should be advised after providing detailed information to the couples [67,68,69,70,71,72].

Another group of patients where the use of IL-1 blockers is controversial includes patients with active malignancy or a history of malignancy in complete remission. The potential effects of the IL-1 pathway blockade on the vulnerability to malignancies have been a matter of apprehension. Nevertheless, contemporary evidence, which is based mainly on the long-lasting experience gained with anakinra, does not support this hypothesis since the observed rates of malignancies were similar to the general population [73,74,75,76,77]. Remarkably, recent data suggest an even protective effect of IL-1 blockade against cancer, such as canakinumab for lung cancer [77]. 

With reference to infections, accumulated data point out that IL-1 inhibition may be associated with an increased risk of infections (especially in patients with rheumatoid arthritis receiving anakinra in combination with glucocorticoids), which, however, are mostly of mild to moderate severity [24,75,78]. Most importantly, compared with glucocorticoids, which are widely administered as a second-step medication in refractory recurrent pericarditis, the risk of severe infections is less prominent [36,78,79,80,81]. Among IL-1 blockers, anakinra, due to its short half-life, should be considered the agent of choice in patients at an increased risk of infections [36]. Finally, chronic kidney disease is not considered a contraindication for IL-1 blocker administration although a close follow-up is recommended in this setting [24]. 

### 4.3. New Treatment Proposals 

Besides colchicine, which is indicated in the whole spectrum of acute and recurrent pericarditis as well as IL-1 blockers that are already considered an established treatment for refractory recurrent pericarditis with an autoinflammatory clinical phenotype, new treatment options have been recently tested in this setting such as those reported below: 

#### 4.3.1. Hydroxychloroquine 

HCQ appears as a promising steroid-sparing agent with a favorable cost–benefit profile [22]. The drug has been recently tested in a pilot observational prospective study including 30 patients (mean age ~49 years, 67% females). All patients were diagnosed with idiopathic colchicine-resistant glucocorticoid-dependent recurrent pericarditis, with autoinflammatory clinical phenotype and a history of at least three recurrences. Among them, 15 received 400 mg of HCQ and the remainder placebo along with the standard-of-care treatment in a non-randomized open-label fashion. All patients were followed up until the first pericarditis flare. Although HCQ did not reduce pericarditis recurrence risk, it has been associated with an increased median time of flare-free survival (increase of 4 weeks), a reduced hazard ratio for flare in survival analysis (HR = 0.36), a higher rate of patients able to halve the dose of glucocorticoids (33.3% vs. 0% in the control group, *p* = 0.037), and lower glucocorticoid dose overall (43.5% vs. control: −4.5%, *p* < 0.001) along with a good safety profile, without any discontinuation of the active drug [22]. Following the promising results observed in this pilot study, a randomized control trial has already been declared. 

It should be emphasized that since hydroxychloroquine is an immunomodulatory agent, an approximately 3-month delay is required until its clinical results become manifest. During this time period, the dose of steroids should not be modified, especially if the dose is close to the threshold where relapses appear and possibly colchicine should be continued, although these patients are considered colchicine-resistant. Moreover, before HCQ administration, an electrocardiogram to check for pre-existing QT-segment prolongation as well as an ophthalmological examination to exclude retinopathy should be performed [22]. 

#### 4.3.2. Beta Blockers

Beta blockers are another class of medications recently tested in patients with recurrent pericarditis. With their property of reducing heart rate, it has been suggested that, on top of standard anti-inflammatory therapies, they may improve symptoms and possibly the risk of recurrence. To test this hypothesis, beta blockers have been administered to 101 symptomatic patients with acute pericarditis and a heart rate >75 bpm. The target heart rate with beta blockade was <70 bpm [23]. A propensity-matched number of control subjects has been enrolled. According to this study’s results, patients receiving beta blockers showed improved symptom control with symptom persistence at 3 weeks of 4% vs. 14% in control subjects, *p* = 0.024. Interestingly, a trend of lower rate of recurrences during follow-up was also recorded in the active medication arm (*p* = 0.069). 

#### 4.3.3. Cannabidiol 

Finally, additional compounds have been recently tested as potential treatments in recurrent pericarditis, such as cannabidiol, a non-psychoactive substance derived from Cannabis Sativa, which possesses anti-inflammatory, vasodilatory, and antioxidant properties [82]. CardiolRxTM (a pure cannabidiol solution), is currently being tested for recurrent pericarditis in the phase II MAvERIC-Pilot Study, which includes 27 patients diagnosed with symptomatic recurrent pericarditis (ClinicalTrials.gov ID NCT05494788). The primary end-point is the patient-reported pericardial pain on an 11-point numerical rating scale at 8 weeks, whereas secondary endpoints include the NRS pain score at 26 weeks and freedom from pericarditis recurrence during an 18-week extension period. Safety and tolerability of the drug are also assessed in this pilot study. 

## 5. Gaps in Knowledge 

As previously mentioned, refractory recurrent pericarditis is a multifaceted disease in terms of severity, duration, response to treatment, and impact on quality of life. For instance, patients with sparse recurrences easily controlled with a course of NSAIDs and colchicine are not actually problematic. On the other hand, patients with glucocorticoid-dependent colchicine-resistant pericarditis needing a high dose of steroids to obtain stable remission may impose a serious concern regarding treatment strategy. Therefore, it is self-explanatory that treatment decisions should be strictly individualized based on the specific clinical scenario. 

In this context, the adoption of the most efficacious treatment protocols with IL-1 blockers is of paramount importance. As already stressed, the IRAP registry clarified that at least 3 months of full dose administration followed by at least 3 months of tapering is associated with the lowest risk of recurrence [54]. Notably, during the first attempts with IL-1 blockers in recurrent pericarditis, the full dose scheme was protracted for up to 12 months and was followed by an abrupt drug discontinuation [8]. However, it soon became clear that this practice was accompanied by an unacceptably high rate of pericarditis recurrence (up to 70%) [83]. This finding highlighted the need for progressive dose reduction similar to glucocorticoids. At present, there is no unanimity about the most suitable anakinra treatment protocol. A widespread protocol proposes anakinra administration for 3–6 months at full dose, followed by the omission of one injection per week every month until discontinuation [36]. 

According to another popular scheme, a full dose is administered for at least 2 months. Then, injections are administered on alternate days, and in the absence of a recurrence, a further dose tapering is attempted with anakinra administration every 3 days for 2 months, then every 4 days for 2 months, and so on [84]. In the case of a relapse, a return to the full dose regimen is performed with a gradual return to the lowest number of injections proved effective during the previous circle of administration. It should be emphasized that in most cases (up to 75%) relapses appear at the time of transition from three to two injections per week [36]. At this point, it is likely that even slower tapering is required. However, even with cautious tapering, long periods of anakinra administration may be required, which in our institutional experience may rarely exceed 10 years. Actually, in a recent study of IL-1 administration (mainly anakinra) in pediatric recurrent pericarditis, during a 24-month follow-up period only ~15% of children were weaned off anakinra [85]. This result is in line with previous data suggesting that the average duration of glucocorticoid-dependent colchicine-resistant recurrent pericarditis is estimated at 4.7 years [27]. Thus, the duration of treatment protocols in refractory cases should be reconsidered. Specifically, it is probably preferable to employ longer-lasting treatment plans with the aim of preventing patients from repeated recurrences. 

In the case of rilonacept, currently, there are no data available with regard to the need for tapering, which is generally not recommended based on the long half-life (~1 week) of the drug. In the long extension arm of RHAPSODY, as mentioned above, 6/8 patients who turned down rilonacept treatment experienced a relapse at a median time of approximately 3 months upon discontinuation [60]. Nevertheless, the discussion of treatment duration with rilonacept and the eventual need for dose tapering is still open. Finally, data for goflikicept administration protocol are very scant. 

Regarding the rest of the medications recently introduced in clinical practice, the protocol of hydroxychloroquine administration is still under investigation [22]. According to our institutional protocol, we recommend continuing the full dose regimen (i.e., 400 mg) for 3 months after glucocorticoid discontinuation and to halve the dose subsequently for an additional 3 months. 

## 6. Future Directions 

Refractory recurrent pericarditis is no longer a difficult ordeal for patients and attending clinicians in the modern era. Indeed, even in desperate cases, it is now possible to provide valuable support to these unfortunate patients. The introduction of IL-1 blockers is a paradigm shift in our approach to the disease. In addition, the exploration of several pathophysiological aspects of recurrent pericarditis has provided a better understanding of the disease [17]. 

Nonetheless, several features of the disease have yet to be addressed further. Specifically, despite their striking efficacy in obtaining sustained pericarditis remission, IL-1 blockers are not able to eliminate the disease; at present, the disease is expected to resolve rather spontaneously at some point in time. During this period of time, the primary target should be to ensure that our patients enjoy the best possible quality of life and are protected against harm attributed to pericarditis medications as much as possible. Thus, in order to inspire confidence in our patients, all the above treatment information, with particular emphasis on the pros and cons of each treatment, should be sufficiently explained. Most importantly, the forthcoming 2025 ESC Guidelines on the management of pericardial diseases are expected to update the contemporary knowledge on pericardial diseases and homogenize the perspectives towards this disorder for the benefit of patients.

## 7. Conclusions 

A better understanding of recurrent pericarditis pathophysiology has contributed to the introduction of new drugs in clinical practice during the last decade, with IL-1 blockers being the most prominent and revolutionary new medications. 

At present, suitable candidates for IL-1 blocker administration include patients with glucocorticoid-dependent colchicine pericarditis with frequent (≥2) relapses who depict an autoinflammatory phenotype (namely CRP elevation, fever, and pleuropulmonary involvement). Additional subsets of patients where IL-1 therapy should be possibly taken into account include contraindications of traditional therapy with NSAIDs and glucocorticoids, such as active peptic ulcer, decompensated heart failure, and advanced chronic kidney disease, among others. 

The most common side effects of IL-1 blocker therapy include, in order of decreasing frequency, transient local skin reactions, increase in transaminases, increased risk of respiratory and skin infections, and reduction in white blood cells. These side effects are usually reversible and some can be prevented by simple measures (such as warming the syringes before use to prevent skin reactions). Prompt recognition of adverse effects is of paramount importance in terms of outcome. 

The accumulation of experience in the genetic architecture of recurrent pericarditis as well as clinical research and real-world data is indispensable in determining the most appropriate use of this class of medications in terms of patient selection, treatment protocols (full dose and tapering process), and prerequisites for discontinuation. In parallel, the ultimate target of intense ongoing pharmacological research is to deliver drugs that not only maintain disease remission but also eliminate recurrent pericarditis. 

## Figures and Tables

**Table 1:** Diagnostic criteria of acute pericarditis (first episode or relapses) and risk factors associated with poor prognosis.

Diagnostic Criteria for Pericarditis (Diagnosis Can Be Made with at Least 2 of the Following Criteria)	High-Risk Criteria Associated with Complicated Pericarditis (Such as Cardiac Tamponade, Arrhythmias, as Well as Recurrence and Constrictive Pericarditis during Follow-Up) and a Specific (Non-Idiopathic and Non-Viral) Etiology
Chest pain worsens with inspiration or lying down and relieved while sitting and leaning forward. ECG showing diffuse concave ST-segment elevation with concomitant PR-segment depression without coronary artery distribution and without new q waves (phase I features). Typical evolution in the following days/weeks with the return of ST-segment to the isoelectric line (phase II), widespread T wave inversion (phase III), and finally ECG normalization (phase IV). Such a series of events is observed in ~50% of cases. Pericardial friction rub (~30%). New-appearing or worsening pericardial effusion (~60% of cases, usually mild).	Major criteria (emerged in multivariate analysis). High fever (38 °C). Subacute presentation of symptoms without a clear-cut onset. Presence of large pericardial effusion (namely >2 cm diastolic echo-free space). Cardiac tamponade. Absence of remission after 1-week treatment with the maximally tolerated dose of anti-inflammatory drugs. Minor criteria (based on experts’ opinions). Myopericarditis (mainly pericarditis with myocardial involvement). Immunodepression. Trauma. Chronic oral anticoagulant therapy.

ECG = electrocardiogram.

**Table 2:** Characteristics and adverse events of IL-1-blockers administered in refractory recurrent pericarditis.

	Anakinra	Rilonacept	Goflikicept
Mechanism of action:	Recombinant human IL-1 receptor antagonist	Dimeric fusion protein that combines two IL-1 receptors with an Fc immunoglobulin tail (IL-1α and 1β trap)	Heterodimeric fusion protein having high affinity for IL-1α and IL-1β
Route of administration:	SC or IV	SC	SC
Biological half-life:	4–6 h	7 days	10 days
Dosing (full dose):	Every day 1–2 mg/kg/day up to 100 mg/day	Once a week Loading: 320 mg on the first day (or 4.4 mg/kg if <18 years of age) Maintenance: 160 mg (or 2.2 mg/kg if <18 years of age)	Every 2 weeks Loading: 160 mg Maintenance: 80 mg on weeks 1 and 2 and then 80 mg
Route of excretion:	Mostly kidney (no dose adjustment is generally required in CKD)	Reticuloendothelial system (no dose adjustment is generally required in CKD)	Not known
Serious side effects requiring drug discontinuation:	3%	3%	4.5%
Main side effects:			
Injection site reactions	38%	33%	18%
Transaminasemia	3%	4%	4.5%
Neutropenia	1%	NR	9.1%
Infections	3%	16%	~23%
Arthralgias/myalgias	6%	12%	NR
Blood lipid elevation	NR	8%	18%
Treatment protocol	At least 3 months full-dose with at least 3 months tapering	At least 6–8 months	No data

SC: subcutaneous, IV: intravenous, CKD = chronic kidney disease, IL = interleukin, and NR = not reported. Data on anakinra side effects were primarily derived from the IRAP registry [24,56], those on rilonacept from the phase II and III (RHAPSODY) clinical trials on recurrent pericarditis [24,57,58], and the relevant data from goflikicept similarly from phase II/III study results [62].

## Data Availability

Data is contained within the article.

## References

[1] Adler Y., Charron P., Imazio M., Badano L., Barón-Esquivias G., Bogaert J., Brucato A., Gueret P., Klingel K., Lionis C. (2015). 2015 ESC Guidelines for the diagnosis and management of pericardial diseases: The Task Force for the Diagnosis and Management of Pericardial Diseases of the European Society of Cardiology (ESC) Endorsed by: The European Association for Cardio-Thoracic Surgery (EACTS). Eur. Heart J..

[2] Vianello F., Cinetto F., Cavraro M., Battisti A., Castelli M., Imbergamo S., Marcolongo R. (2011). Azathioprine in isolated recurrent pericarditis: A single centre experience. Int. J. Cardiol..

[3] Imazio M., Lazaros G., Picardi E., Vasileiou P., Carraro M., Tousoulis D., Belli R., Gaita F. (2016). Intravenous human immunoglobulins for refractory recurrent pericarditis: A systematic review of all published cases. J. Cardiovasc. Med..

[4] Picco P., Brisca G., Traverso F., Loy A., Gattorno M., Martini A. (2009). Successful treatment of idiopathic recurrent pericarditis in children with interleukin-1β receptor antagonist (anakinra): An unrecognized autoinflammatory disease?. Arthritis Rheum..

[5] Lopalco G., Rigante D., Cantarini L., Imazio M., Lopalco A., Emmi G., Venerito V., Fornaro M., Frediani B., Nivuori M. (2021). The autoinflammatory side of recurrent pericarditis: Enlightening the pathogenesis for a more rational treatment. Trends Cardiovasc. Med..

[6] Peet C.J., Rowczenio D., Omoyinmi E., Papadopoulou C., Mapalo B.R.R., Wood M.R., Capon F., Lachmann H.J. (2022). Pericarditis and Autoinflammation: A Clinical and Genetic Analysis of Patients with Idiopathic Recurrent Pericarditis and Monogenic Autoinflammatory Diseases at a National Referral Center. J. Am. Heart Assoc..

[7] Finetti M., Insalaco A., Cantarini L., Meini A., Breda L., Alessio M., D’Alessandro M., Picco P., Martini A., Gattorno M. (2014). Long-Term Efficacy of Interleukin-1 Receptor Antagonist (Anakinra) in Corticosteroid-Dependent and Colchicine-Resistant Recurrent Pericarditis. J. Pediatr..

[8] Vassilopoulos D., Lazaros G., Tsioufis C., Vasileiou P., Stefanadis C., Pectasides D. (2012). Successful treatment of adult patients with idiopathic recurrent pericarditis with an interleukin-1 receptor antagonist (anakinra). Int. J. Cardiol..

[9] Brucato A., Imazio M., Cremer P.C., Adler Y., Maisch B., Lazaros G., Gattorno M., Caforio A.L.P., Marcolongo R., Emmi G. (2018). Recurrent pericarditis: Still idiopathic? The pros and cons of a well-honoured term. Intern. Emerg. Med..

[10] Cantarini L., Lucherini O.M., Brucato A., Barone L., Cumetti D., Iacoponi F., Rigante D., Brambilla G., Penco S., Brizi M.G. (2012). Clues to detect tumor necrosis factor receptor-associated periodic syndrome (TRAPS) among patients with idiopathic recurrent acute pericarditis: Results of a multicentre study. Clin. Res. Cardiol..

[11] Cinteza E., Stefan D., Iancu M.A., Ioan A., Vasile C.M., Vatasescu R., Cochino A. (2023). Autoinflammatory Recurrent Pericarditis Associated with a New NLRP12 Mutation in a Male Adolescent. Life.

[12] Toldo S., Mezzaroma E., Buckley L.F., Potere N., Di Nisio M., Biondi-Zoccai G., Van Tassell B.W., Abbate A. (2022). Targeting the NLRP3 inflammasome in cardiovascular diseases. Pharmacol. Ther..

[13] Del Buono M.G., Bonaventura A., Vecchié A., Moroni F., Golino M., Bressi E., De Ponti R., Dentali F., Montone R.A., Kron J. (2023). Pathogenic pathways and therapeutic targets of inflammation in heart diseases: A focus on Interleukin-1. Eur. J. Clin. Investig..

[14] Toldo S., Abbate A. (2023). The role of the NLRP3 inflammasome and pyroptosis in cardiovascular diseases. Nat. Rev. Cardiol..

[15] Abbate A., Toldo S., Marchetti C., Kron J., Van Tassell B.W., Dinarello C.A. (2020). Interleukin-1 and the Inflammasome as Therapeutic Targets in Cardiovascular Disease. Circ. Res..

[16] Imazio M., Mardigyan V., Andreis A., Franchin L., De Biasio M., Collini V. (2023). New Developments in the Management of Recurrent Pericarditis. Can. J. Cardiol..

[17] Dong T., Klein A.L., Wang T.K.M. (2023). Paradigm Shift in Diagnosis and Targeted Therapy in Recurrent Pericarditis. Curr. Cardiol. Rep..

[18] Thomas G.K., Bonaventura A., Vecchié A., van Tassell B., Imazio M., Klein A., Luis S.A., Abbate A. (2024). Interleukin-1 Blockers for the Treatment of Recurrent Pericarditis: Pathophysiology, Patient-Reported Outcomes, and Perspectives. J. Cardiovasc. Pharmacol..

[19] Vecchié A., Del Buono M.G., Mauro A.G., Cremer P.C., Imazio M., Klein A.L., Abbate A., Dentali F., Bonaventura A. (2022). Advances in pharmacotherapy for acute and recurrent pericarditis. Expert Opin. Pharmacother..

[20] Bonaventura A. (2022). The long journey of interleukin-1 in acute and recurrent pericarditis. Eur. Heart J..

[21] Kumar S., Khubber S., Reyaldeen R., Agrawal A., Cremer P.C., Imazio M., Kwon D.H., Klein A.L. (2022). Advances in Imaging and Targeted Therapies for Recurrent Pericarditis: A Review. JAMA Cardiol..

[22] Lazaros G., Antonopoulos A.S., Antonatou K., Skendros P., Ritis K., Hadziyannis E., Lazarou E., Leontsinis I., Simantiris S., Vlachopoulos C. (2020). Hydroxychloroquine for colchicine-resistant glucocorticoid-dependent idiopathic recurrent pericarditis: A pilot observational prospective study. Int. J. Cardiol..

[23] Imazio M., Andreis A., Agosti A., Piroli F., Avondo S., Casula M., Paneva E., Squarotti G.B., Giustetto C., De Ferrari G.M. (2021). Usefulness of Beta-Blockers to Control Symptoms in Patients with Pericarditis. Am. J. Cardiol..

[24] Imazio M., Lazaros G., Gattorno M., LeWinter M., Abbate A., Brucato A., Klein A. (2021). Anti-interleukin-1 agents for pericarditis: A primer for cardiologists. Eur. Heart J..

[25] Cremer P.C., Kumar A., Kontzias A., Tan C.D., Rodriguez E.R., Imazio M., Klein A.L. (2016). Complicated Pericarditis: Understanding Risk Factors and Pathophysiology to Inform Imaging and Treatment. J. Am. Coll. Cardiol..

[26] Andreis A., Imazio M., Giustetto C., Brucato A., Adler Y., De Ferrari G.M. (2020). Anakinra for constrictive pericarditis associated with incessant or recurrent pericarditis. Heart.

[27] Brucato A., Brambilla G., Moreo A., Alberti A., Munforti C., Ghirardello A., Doria A., Shinar Y., Livneh A., Adler Y. (2006). Long-Term Outcomes in Difficult-to-Treat Patients with Recurrent Pericarditis. Am. J. Cardiol..

[28] Collini V., Vignut L.S., Angriman F., Braidotti G., De Biasio M., Imazio M. (2024). Age-stratified patterns in clinical presentation, treatment and outcomes in acute pericarditis: A retrospective cohort study. Heart.

[29] Collini V., Andreis A., De Biasio M., De Martino M., Isola M., Croatto N., Lepre V., Cantarini L., Merlo M., Sinagra G. (2024). Efficacy of colchicine in addition to anakinra in patients with recurrent pericarditis. Open Heart.

[30] LeWinter M.M. (2014). Clinical practice. Acute Pericarditis. N. Engl. J. Med..

[31] Khandaker M.H., Espinosa R.E., Nishimura R.A., Sinak L.J., Hayes S.N., Melduni R.M., Oh J.K. (2010). Pericardial Disease: Diagnosis and Management. Mayo Clin. Proc..

[32] Lilly L.S. (2013). Treatment of Acute and Recurrent Idiopathic Pericarditis. Circulation.

[33] Imazio M., Demichelis B., Parrini I., Giuggia M., Cecchi E., Gaschino G., Demarie D., Ghisio A., Trinchero R. (2004). Day-hospital treatment of acute pericarditis: A management program for outpatient therapy. J. Am. Coll. Cardiol..

[34] Imazio M., Andreis A., Lubian M., Lazaros G., Lazarou E., Brucato A., Adler Y., Giustetto C., Rinaldi M., De Ferrari G.M. (2021). The Torino Pericarditis Score: A new-risk stratification tool to predict complicated pericarditis. Intern. Emerg. Med..

[35] Lazarou E., Lazaros G., Antonopoulos A.S., Imazio M., Vasileiou P., Karavidas A., Toutouzas K., Vassilopoulos D., Tsioufis C., Tousoulis D. (2021). A risk score for pericarditis recurrence. Eur. J. Clin. Investig..

[36] Lazarou E., Koutsianas C., Theofilis P., Lazaros G., Vassilopoulos D., Vlachopoulos C., Tsioufis C., Imazio M., Brucato A., Tousoulis D. (2024). Interleukin-1 Blockers: A Paradigm Shift in the Treatment of Recurrent Pericarditis. Life.

[37] Imazio M., Spodick D.H., Brucato A., Trinchero R., Adler Y. (2010). Controversial Issues in the Management of Pericardial Diseases. Circulation.

[38] Khandaker M.H., Schaff H.V., Greason K.L., Anavekar N.S., Espinosa R.E., Hayes S.N., Nishimura R.A., Oh J.K. (2012). Pericardiectomy vs. Medical Management in Patients with Relapsing Pericarditis. Mayo Clin. Proc..

[39] McGonagle D., McDermott M.F. (2006). A Proposed Classification of the Immunological Diseases. PLoS Med..

[40] Caforio A.L.P., Brucato A., Doria A., Brambilla G., Angelini A., Ghirardello A., Bottaro S., Tona F., Betterle C., Daliento L. (2010). Anti-heart and anti-intercalated disk autoantibodies: Evidence for autoimmunity in idiopathic recurrent acute pericarditis. Heart.

[41] Lazaros G., Antonatou K., Vassilopoulos D. (2017). The Therapeutic Role of Interleukin-1 Inhibition in Idiopathic Recurrent Pericarditis: Current Evidence and Future Challenges. Front. Med..

[42] Tombetti E., Giani T., Brucato A., Cimaz R. (2019). Recurrent Pericarditis in Children and Adolescents. Front. Pediatr..

[43] Swanson K.V., Deng M., Ting J.P.-Y. (2019). The NLRP3 inflammasome: Molecular activation and regulation to therapeutics. Nat. Rev. Immunol..

[44] Hardy R.S., Raza K., Cooper M.S. (2020). Therapeutic glucocorticoids: Mechanisms of actions in rheumatic diseases. Nat. Rev. Rheumatol..

[45] Imazio M., Brucato A., Cemin R., Ferrua S., Maggiolini S., Beqaraj F., Demarie D., Forno D., Ferro S., Maestroni S. (2013). A Randomized Trial of Colchicine for Acute Pericarditis. N. Engl. J. Med..

[46] Leung Y.Y., Yao Hui L.L., Kraus V.B. (2015). Colchicine—Update on mechanisms of action and therapeutic uses. Semin. Arthritis Rheum..

[47] Schrezenmeier E., Dörner T. (2020). Mechanisms of action of hydroxychloroquine and chloroquine: Implications for rheumatology. Nat. Rev. Rheumatol..

[48] Imazio M. (2020). Noninfectious pericarditis: Management challenges for cardiologists. Kardiologia Polska.

[49] De La Serna A., Soldevila J., Claramunt V., De Luna A. (1987). Colchicine for recurrent pericarditis. Lancet.

[50] Bayes-Genis A., Adler Y., de Luna A.B., Imazio M. (2017). Colchicine in Pericarditis. Eur. Heart J..

[51] Imazio M., Bobbio M., Cecchi E., Demarie D., Pomari F., Moratti M., Ghisio A., Belli R., Trinchero R. (2005). Colchicine as first-choice therapy for recurrent pericarditis: Results of the CORE (COlchicine for REcurrent pericarditis) trial. Arch. Intern. Med..

[52] Imazio M., Brucato A., Cemin R., Ferrua S., Belli R., Maestroni S., Trinchero R., Spodick D.H., Adler Y. (2011). Colchicine for recurrent pericarditis (CORP): A randomized trial. Ann. Intern. Med..

[53] Imazio M., Belli R., Brucato A., Cemin R., Ferrua S., Beqaraj F., Demarie D., Ferro S., Forno D., Maestroni S. (2014). Efficacy and safety of colchicine for treatment of multiple recurrences of pericarditis (CORP-2): A multicentre, double-blind, placebo-controlled, randomised trial. Lancet.

[54] Cohen S.B. (2004). The use of anakinra, an interleukin-1 receptor antagonist, in the treatment of rheumatoid arthritis. Rheum. Dis. Clin. N. Am..

[55] Brucato A., Imazio M., Gattorno M., Lazaros G., Maestroni S., Carraro M., Finetti M., Cumetti D., Carobbio A., Ruperto N. (2016). Effect of Anakinra on Recurrent Pericarditis among Patients with Colchicine Resistance and Corticosteroid Dependence: The AIRTRIP Randomized Clinical Trial. JAMA.

[56] Imazio M., Andreis A., De Ferrari G.M., Cremer P.C., Mardigyan V., Maestroni S., Luis S.A., Lopalco G., Emmi G., Lotan D. (2020). Anakinra for corticosteroid-dependent and colchicine-resistant pericarditis: The IRAP (International Registry of Anakinra for Pericarditis) study. Eur. J. Prev. Cardiol..

[57] Klein A.L., Lin D., Cremer P.C., Nasir S., Luis S.A., Abbate A., Ertel A., LeWinter M., Beutler A., Fang F. (2020). Efficacy and safety of rilonacept for recurrent pericarditis: Results from a phase II clinical trial. Heart.

[58] Klein A.L., Imazio M., Cremer P., Brucato A., Abbate A., Fang F., Insalaco A., LeWinter M., Lewis B.S., Lin D. (2021). Phase 3 Trial of Interleukin-1 Trap Rilonacept in Recurrent Pericarditis. N. Engl. J. Med..

[59] Brucato A., Lim-Watson M.Z., Klein A., Imazio M., Cella D., Cremer P., LeWinter M.M., Luis S.A., Lin D., Lotan D. (2022). Interleukin-1 Trap Rilonacept Improved Health-Related Quality of Life and Sleep in Patients with Recurrent Pericarditis: Results from the Phase 3 Clinical Trial RHAPSODY. J. Am. Heart Assoc..

[60] Imazio M., Klein A.L., Brucato A., Abbate A., Arad M., Cremer P.C., Insalaco A., LeWinter M.M., Lewis B.S., Lin D. (2024). Sustained Pericarditis Recurrence Risk Reduction with Long-Term Rilonacept. J. Am. Heart Assoc..

[61] Imazio M., Andreis A., Piroli F., Lazaros G., Gattorno M., Lewinter M., Klein A.L., Brucato A. (2021). Anti-interleukin 1 agents for the treatment of recurrent pericarditis: A systematic review and meta-analysis. Heart.

[62] Myachikova V.Y., Maslyanskiy A.L., Moiseeva O.M., Vinogradova O.V., Gleykina E.V., Lavrovsky Y., Abbate A., Grishin S.A., Egorova A.N., Schedrova M.L. (2023). Treatment of Idiopathic Recurrent Pericarditis with Goflikicept: Phase II/III Study Results. J. Am. Coll. Cardiol..

[63] Ridker P.M., Everett B.M., Thuren T., MacFadyen J.G., Chang W.H., Ballantyne C., Fonseca F., Nicolau J., Koenig W., Anker S.D. (2017). Antiinflammatory Therapy with Canakinumab for Atherosclerotic Disease. N. Engl. J. Med..

[64] Kougkas N., Fanouriakis A., Papalopoulos I., Bertsias G., Avgoustidis N., Repa A., Sidiropoulos P. (2018). Canakinumab for recurrent rheumatic disease associated-pericarditis: A case series with long-term follow-up. Rheumatology.

[65] Epçaçan S., Sahin S., Kasapcopur O. (2019). Anaphylactic reaction to anakinra in a child with steroid-dependent idiopathic recurrent pericarditis and successful management with canakinumab. Cardiol. Young.

[66] Signa S., D’alessandro M., Consolini R., Miniaci A., Bustaffa M., Longo C., Tosca M.A., Bizzi M., Caorsi R., Mendonça L.O. (2020). Failure of anti Interleukin-1 β monoclonal antibody in the treatment of recurrent pericarditis in two children. Pediatr. Rheumatol..

[67] Chang Z., Spong C.Y., Jesus A.A., Davis M.A., Plass N., Stone D.L., Chapelle D., Hoffmann P., Kastner D.L., Barron K. (2014). Brief Report: Anakinra Use during Pregnancy in Patients with Cryopyrin-Associated Periodic Syndromes (CAPS). Arthritis Rheumatol..

[68] Russell M.D., Dey M., Flint J., Davie P., Allen A., Crossley A., Frishman M., Gayed M., Hodson K., Khamashta M. (2023). British Society for Rheumatology guideline on prescribing drugs in pregnancy and breastfeeding: Immunomodulatory anti-rheumatic drugs and corticosteroids. Rheumatology.

[69] Youngstein T., Hoffmann P., Gül A., Lane T., Williams R., Rowczenio D.M., Ozdogan H., Ugurlu S., Ryan J., Harty L. (2017). International multi-centre study of pregnancy outcomes with interleukin-1 inhibitors. Rheumatology.

[70] Smith C.J.F., Chambers C.D. (2018). Five successful pregnancies with antenatal anakinra exposure. Rheumatology.

[71] Brien M.-E., Gaudreault V., Hughes K., Hayes D.J.L., Heazell A.E.P., Girard S. (2021). A Systematic Review of the Safety of Blocking the IL-1 System in Human Pregnancy. J. Clin. Med..

[72] Sammaritano L.R., Bermas B.L., Chakravarty E.E., Chambers C., Clowse M.E.B., Lockshin M.D., Marder W., Guyatt G., Branch D.W., Buyon J. (2020). 2020 American College of Rheumatology Guideline for the Management of Reproductive Health in Rheumatic and Musculoskeletal Diseases. Arthritis Care Res..

[73] Wu T.-C., Xu K., Martinek J., Young R.R., Banchereau R., George J., Turner J., Kim K.I., Zurawski S., Wang X. (2018). IL1 Receptor Antagonist Controls Transcriptional Signature of Inflammation in Patients with Metastatic Breast Cancer. Cancer Res..

[74] Xie W., Xiao S., Huang Y., Sun X., Gao D., Ji L., Li G., Zhang Z. (2020). A meta-analysis of biologic therapies on risk of new or recurrent cancer in patients with rheumatoid arthritis and a prior malignancy. Rheumatology.

[75] Fleischmann R.M., Tesser J., Schiff M.H., Schechtman J., Burmester G.-R., Bennett R., Modafferi D., Zhou L., Bell D., Appleton B. (2006). Safety of extended treatment with anakinra in patients with rheumatoid arthritis. Ann. Rheum. Dis..

[76] Xie J., Zhang Y., Jiang L. (2022). Role of Interleukin-1 in the pathogenesis of colorectal cancer: A brief look at anakinra therapy. Int. Immunopharmacol..

[77] Ridker P.M., MacFadyen J.G., Thuren T., Everett B.M., Libby P., Glynn R.J., Ridker P., Lorenzatti A., Krum H., Varigos J. (2017). Effect of interleukin-1β inhibition with canakinumab on incident lung cancer in patients with atherosclerosis: Exploratory results from a randomised, double-blind, placebo-controlled trial. Lancet.

[78] Spera A.M. (2022). Hepatitis B virus infection reactivation in patients under immunosuppressive therapies: Pathogenesis, screening, prevention and treatment. World J. Virol..

[79] Kullenberg T., Löfqvist M., Leinonen M., Goldbach-Mansky R., Olivecrona H. (2016). Long-term safety profile of anakinra in patients with severe cryopyrin-associated periodic syndromes. Rheumatology.

[80] Anakinra (2012). LiverTox: Clinical and Research Information on Drug-Induced Liver Injury.

[81] Winthrop K., Mariette X., Silva J., Benamu E., Calabrese L., Dumusc A., Smolen J., Aguado J., Fernández-Ruiz M. (2018). ESCMID Study Group for Infections in Compromised Hosts (ESGICH) Consensus Document on the safety of targeted and biological therapies: An infectious diseases perspective (Soluble immune effector molecules [II]: Agents targeting interleukins, immunoglobulins and complement factors). Clin. Microbiol. Infect..

[82] Naya N.M., Kelly J., Hogwood A., Abbate A., Toldo S. (2024). Therapeutic potential of cannabidiol (CBD) in the treatment of cardiovascular diseases. Expert Opin. Investig. Drugs.

[83] Lazaros G., Vasileiou P., Koutsianas C., Antonatou K., Stefanadis C., Pectasides D., Vassilopoulos D. (2014). Anakinra for the management of resistant idiopathic recurrent pericarditis. Initial experience in 10 adult cases: Table 1. Ann. Rheum. Dis..

[84] Aldajani A., Imazio M., Klein A., Mardigyan V. (2023). How to Use Interleukin-1 Antagonists in Patients with Pericarditis. Can. J. Cardiol..

[85] Caorsi R., Insalaco A., Bovis F., Martini G., Cattalini M., Chinali M., Rimini A., Longo C., Federici S., Celani C. (2023). Pediatric Recurrent Pericarditis: Appropriateness of the Standard of Care and Response to IL-1 Blockade. J. Pediatr..

